# Multidisciplinary Practical Guidance for Implementing Adjuvant CDK4/6 Inhibitors for Patients with HR-Positive, HER2-Negative Early Breast Cancer in Canada

**DOI:** 10.3390/curroncol32080444

**Published:** 2025-08-07

**Authors:** Katarzyna J. Jerzak, Sandeep Sehdev, Jean-François Boileau, Christine Brezden-Masley, Nadia Califaretti, Scott Edwards, Jenn Gordon, Jan-Willem Henning, Nathalie LeVasseur, Cindy Railton

**Affiliations:** 1 Division of Medical Oncology, Sunnybrook Odette Cancer Centre, University of Toronto, Toronto, ON M4N 3M5, Canada; 2 Division of Medical Oncology, The Ottawa Hospital Cancer Centre, Ottawa, ON K1H 8L6, Canada; 3 Department of Surgery, Jewish General Hospital, Montreal, QC H3T 1E2, Canada; 4 Department of Medical Oncology, Mount Sinai Hospital, Toronto, ON M5G 1X5, Canada; 5 Grand River Regional Cancer Centre, Kitchener, ON N2G 1G3, Canada; nadia.califaretti@grhosp.on.ca; 6 Dr. H. Bliss Murphy Cancer Centre, St. John’s, NL A1B 3V6, Canada; 7 Rethink Breast Cancer, Toronto, ON M4M 3G3, Canada; 8 Department of Oncology, University of Calgary, Calgary, AB T2N 1N4, Canada; 9 Department of Medical Oncology, BC Cancer, Vancouver, BC V5Z 4E6, Canada; nathalie.levasseur@bccancer.bc.ca

**Keywords:** CDK4/6 inhibitors, HR+, HER2− early breast cancer, adjuvant therapy, multidisciplinary care, practical recommendations

## Abstract

Cyclin-dependent kinase (CDK)4/6 inhibitors have become a key component of adjuvant treatment for patients with hormone receptor-positive (HR+), human epidermal growth factor receptor 2-negative (HER2−) early breast cancer who are at high risk of recurrence. The addition of abemaciclib and ribociclib to standard endocrine therapy has demonstrated clinically meaningful improvements in invasive disease-free survival, supported by the monarchE and NATALEE trials, respectively. With expansion of patient eligibility for CDK4/6 inhibitors, multidisciplinary coordination among medical oncologists, surgeons, nurses, pharmacists, and other health care providers is critical to optimizing patient identification, monitoring, and management of adverse events. This expert guidance document provides practical recommendations for implementing adjuvant CDK4/6 inhibitor therapy in routine clinical practice, incorporating insights from multiple specialties and with patient advocacy representation. Key considerations include patient selection based on clinical trial data, treatment duration, dosing schedules, adverse event profiles, monitoring requirements, drug–drug interactions, and patient-specific factors such as tolerability, cost, and quality of life. This guidance aims to support Canadian clinicians in effectively integrating CDK4/6 inhibitors into clinical practice, ensuring optimal patient outcomes through a multidisciplinary and patient-centric approach.

## Simple Summary

Breast cancer is one of the most common cancers, and some patients remain at risk of recurrence even after surgery and hormone therapy. Treatment with cyclin-dependent kinase 4/6 inhibitors, when added to standard hormone treatment, can further reduce this risk. Clinical studies with two of these treatments, abemaciclib and ribociclib, have shown improved outcomes for patients at higher risk of recurrence. As more patients may soon be eligible for these treatments, coordinated care among medical oncologists, surgeons, nurses, and pharmacists is essential to select appropriate patients, monitor for side effects, and support treatment adherence. This article offers expert recommendations on integrating cyclin-dependent kinase 4/6 inhibitors into routine practice, including patient selection, side effect management, and consideration of individual factors such as other medications and quality of life. The goal is to help Canadian healthcare teams implement these therapies effectively and equitably, ensuring patients benefit fully from advances in breast cancer care.

## 1. Introduction

The risk of breast cancer recurrence among patients with early-stage hormone receptor-positive (HR+), human epidermal growth factor receptor 2-negative (HER2−) disease is high, particularly among patients with lymph node-positive disease, where 1 in 6 experience recurrence or death within 5 years of initiating endocrine therapy (ET) [1]. In the past, the management of patients with early-stage HR+ disease involved use of adjuvant ET with the addition of ovarian function suppression for pre-/peri-menopausal patients to reduce the risk of disease recurrence and death [1,2,3]. Despite this approach, the risk of distant recurrence remains high both initially and beyond the first 5 years of treatment [2]. An analysis of 155,746 women with estrogen receptor-positive (ER+) early-stage breast cancer who were scheduled to receive five years of ET demonstrated a 10-year distant recurrence risk of 8%, 15%, and 30% for patients with zero, one to three, and four to nine positive nodes, respectively, during the years 2000–09 [4]. Given this significant risk of recurrence for patients with node-positive disease, it is critical to ensure that they receive appropriate treatment to minimize this risk.

Although the definition of high-risk breast cancer continues to evolve, key features associated with a high risk of breast cancer recurrence include: younger age at initial diagnosis [5,6,7,8], premenopausal status [5], Ki-67 ≥ 20% [9], tumour size ≥ 5 cm (T3) or tumour of any size with direct extension to chest wall, skin, or both (T4a-d) [9], high tumour grade (3) [9], and nodal involvement [9]. Real-world data further highlight that patients with high-risk N1 disease (grade 3 and/or tumour ≥ 5 cm) demonstrate a 2.2-fold increased risk of recurrence compared to patients with N1 disease without these high-risk features [10]. Ultimately, an integrated assessment of clinical and pathological factors provides a comprehensive framework for identifying patients at high risk of disease recurrence [11]. Additionally, complementary genomic tools such as Oncotype DX [12,13] or MammaPrint^®^ [14] can help refine recurrence risk to enhance decision-making [14].

More recently, cyclin-dependent kinase (CDK)4/6 inhibitors have emerged as a cornerstone in breast cancer management and are recommended as adjuvant treatment for patients with HR+, HER2− early breast cancer who are considered at high risk for disease recurrence. In the metastatic setting, three CDK4/6 inhibitors—palbociclib, ribociclib, and abemaciclib—are approved as first- and second-line treatment options [15,16,17,18]. CDK4/6 inhibitors have also demonstrated improved invasive disease-free survival (iDFS) in patients with HR+, HER2− early breast cancer [19,20]. Both abemaciclib (monarchE) and ribociclib (NATALEE) demonstrated positive results in clinical trials for high-risk early breast cancer [19,20]. In contrast, although palbociclib is approved for metastatic breast cancer, it failed to show benefit in the adjuvant setting for early breast cancer in the PALLAS and PENELOPE-B trials [4,21].

Although both monarchE and NATALEE enrolled patients with early-stage HR+, HER2− breast cancer at increased risk of recurrence, the eligibility criteria differed [19,20]. monarchE focused on patients with node-positive disease and additional high-risk clinical-pathologic features (e.g., tumour size ≥ 5 cm, grade 3, or Ki-67 ≥ 20%) [19], while NATALEE included a broader population, i.e., patients with stage IIA disease and high genomic risk, even in the absence of nodal involvement [20]. A detailed comparison of eligibility criteria is provided later in the manuscript.

These distinctions directly impact clinical practice by expanding the pool of patients eligible for adjuvant CDK4/6 inhibitors, which is expected to place additional demands on healthcare systems due to the need for accurate patient identification, regular follow-ups, and need for supportive care throughout the patient journey. This reinforces the need for multidisciplinary collaboration of care teams involving medical oncologists, surgeons, nurses, and pharmacists to effectively manage clinic workload and optimize patient care [22]. Accordingly, shared care models, such as those involving nurse practitioners (NPs), pharmacists, or primary care providers, are being explored to help alleviate pressure on oncology services but will need to be tailored to local infrastructure and resources [23,24]. Optimizing both multidisciplinary collaboration and shared care delivery will be essential to ensure that CDK4/6 inhibitors can be implemented effectively and equitably, recognizing that a one-size-fits-all approach is unlikely to be feasible across medical institutions.

With this, optimizing patient identification, monitoring, and adverse event (AE) management remains essential. This expert opinion review aims to provide practical guidance for implementing adjuvant CDK4/6 inhibitor treatment for patients with HR+, HER2− early-stage breast cancer, incorporating multidisciplinary perspectives from medical and surgical oncologists, a nurse practitioner (NP), an oncology pharmacist, and a patient group representative to maximize successful use of these therapies in Canada.

## 2. Patient Identification for Adjuvant CDK4/6 Inhibitor Therapy

### 2.1. Expert Recommendations for Patient Identification

Adjuvant CDK4/6 inhibitor eligibility should be determined using clinical staging at presentation and pathological features after curative resection. For patients being considered for neoadjuvant therapy, Ki-67 testing should ideally be performed on the core biopsy before systemic treatment to reflect the unaltered disease state. Sentinel lymph node biopsy (SLNB) is the primary method for axillary staging for patients with limited nodal disease [25], providing adequate prognostic information while minimizing the risks of lymphedema and morbidity associated with axillary lymph node dissection (ALND) [26,27]. ALND remains necessary for staging in cases where the accuracy of SLNB is unproven, for maintaining local control in patients with a significant axillary tumour burden, and where knowledge of the extent of nodal involvement alters the choice of systemic therapy [25]. However, routine axillary dissection is generally unnecessary for patients with positive sentinel nodes in the adjuvant setting, as upstaging from N1 to N2 is uncommon, occurring in 2–15% of cases [26,28].

Recent data from the SOUND and INSEMA trials have questioned the necessity of SLNB in select patients with early-stage, clinically node-negative breast cancer [29,30]. Neither trial showed significant differences in local/regional control, iDFS, distant recurrence or overall survival between SLNB and observation, despite approximately 10% of patients in the no-SLNB arms having occult nodal metastases [29,30]. These findings support the selective omission of SLNB in patients with low-risk, node-negative disease, particularly those with HR+, HER2− breast cancer. However, several factors limit generalizability to higher-risk groups. Both trials required axillary ultrasound, reinforcing its importance for patient selection [29,30]. In addition, the enrolled populations were predominantly low-risk, with most patients having small (cT1), low-grade (grade 1–2) tumours, and receiving whole-breast radiation; partial breast irradiation was not permitted, and data on regional nodal radiation were not reported [29,30]. As CDK4/6 inhibitors are indicated for patients with high-risk features, caution is warranted in extrapolating these findings to those with larger or grade 3 tumours. For patients with cN0 and intermediate-risk profiles (e.g., pT2, grade 2), more routine use of genomic assays such as Oncotype DX or MammaPrint^®^ may support appropriate systemic treatment decisions and ensure patients are not excluded from potentially beneficial adjuvant therapies.

In the NATALEE trial, ALND was preferred for nodal staging, but SLNB was permitted as an alternative, and the trial included patients with both node-positive and high-risk node-negative disease [31]. In contrast, the monarchE trial required patients to have node-positive, high-risk early breast cancer, establishing nodal burden as a critical determinant of eligibility. While accurate nodal staging was essential for patient selection in monarchE, identifying extensive nodal disease (e.g., N2 or higher) may be less relevant for ribociclib use, as additional high-risk criteria for patients with node-positive disease were not required in the NATALEE trial [31]. In the Canadian context, eligibility for adjuvant CDK4/6 inhibitors will ultimately be shaped by regulatory approval and reimbursement decisions; however, careful pre-operative assessment, including clinical exam and axillary ultrasound, remains essential to guide surgical planning and inform treatment decisions.

Ultimately, we recommend eligibility for adjuvant CDK4/6 inhibitor therapy be based on an individualized risk assessment incorporating clinical and pathological features, supplemented by gene expression profiling. Furthermore, patient eligibility criteria should align with the intent-to-treat (ITT) population of the monarchE (i.e., cohort 1 and 2) and/or NATALEE trials (Table 1) [19,20].

### 2.2. Optimizing Multidisciplinary Collaboration

At initial assessment, medical oncologists should evaluate each individual patient’s risk of recurrence and determine eligibility for adjuvant CDK4/6 inhibitor therapy. Pharmacists should provide input on potential drug interactions or contraindications that may influence treatment decisions. Furthermore, nurses, NPs, and pharmacists play supportive roles in patient education, treatment discussions with patients, AE management, addressing any safety concerns, and assessing treatment adherence. For cases with conflicting prognostic signals, relative contraindications or drug interactions, and/or uncertainty around the choice of CDK4/6 inhibitor, we recommend collaborative decision-making through multidisciplinary team reviews, with tumour boards reserved for complex cases.

### 2.3. Practical Recommendations for Shared Decision-Making with Patients

Effective communication and planning are essential when guiding patients through the complex and often prolonged treatment journey for HR+, HER2− early breast cancer. Given the already poor adherence to ET in patients with breast cancer, it is crucial for higher-risk populations to strictly follow their treatment plans [41]. Patients may struggle with processing multiple treatment steps simultaneously, so it is helpful to adopt a stepwise counselling approach, with early introduction of subsequent treatments like adjuvant CDK4/6 inhibitors as standard of care agents. CDK4/6 inhibitors can be described to patients as “partner agents” to ET, which can help reduce their risk of disease recurrence. Additionally, reassuring patients that many AEs of CDK4/6 inhibitors can be effectively managed may increase their willingness to initiate treatment. During patient counselling, it is important to use plain language and tailored messages to help patients grasp the relative benefits and risks of CDK4/6 inhibitors. Incorporating tools such as visual aids, videos, and/or summaries can help foster understanding of this complex topic and improve psychological readiness and patient adherence.

## 3. Considerations for Selecting CDK4/6 Inhibitors

### 3.1. Expert Recommendations for CDK4/6 Inhibitor Selection

Selecting the right CDK4/6 inhibitor requires evaluation of trial eligibility, data maturity, treatment duration, dosing and administration, AE profiles, monitoring needs, drug interactions, and patient-specific factors such as tolerability, access, cost, and quality of life. A summary of key differences between the two CDK4/6 inhibitors that have shown efficacy in the adjuvant setting, abemaciclib and ribociclib, is presented in Table 1.

**Figure 1:**
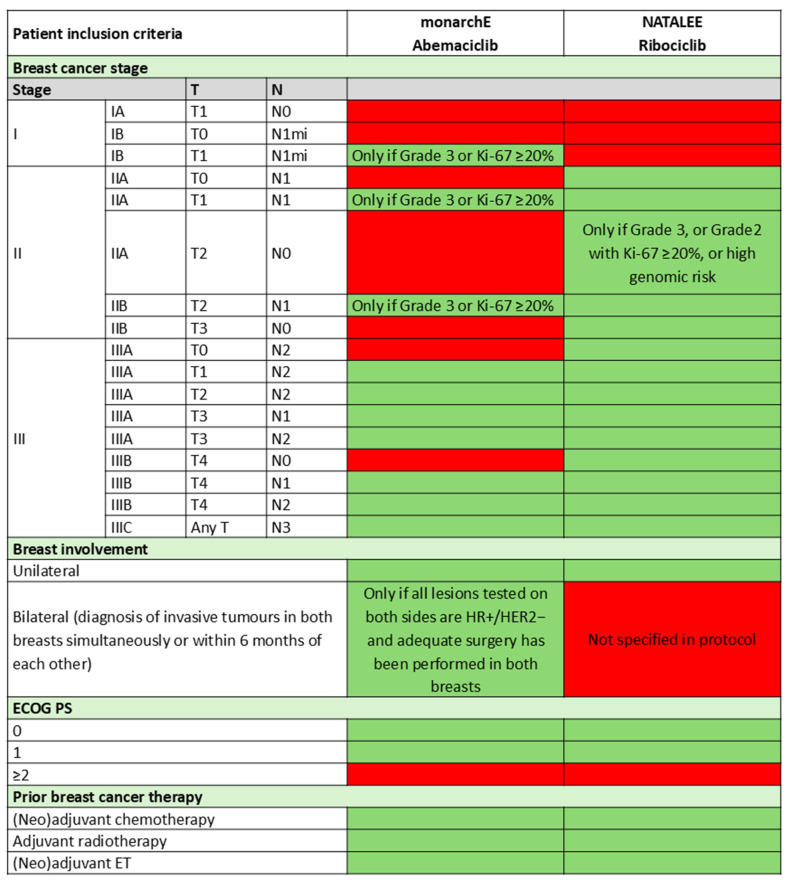
Patient Inclusion Criteria for monarchE and NATALEE [31,43,44]. Green indicates patient eligibility and red indicates patient ineligibility. ECOG: Eastern Cooperative Oncology Group; ET: endocrine therapy; HER2: human epidermal growth factor receptor 2; HR: hormone receptor; mi: micrometastasis; PS: performance status. Green indicates part of inclusion criteria and red indicates not part of inclusion criteria.

Overall, eligibility criteria for monarchE (abemaciclib) and NATALEE (ribociclib) should guide treatment selection, with key differences in eligibility criteria for the trials summarized in Figure 1. For patients meeting criteria for both CDK4/6 inhibitors, our recommendation is aligned with the latest 2024 ASCO guideline update, favouring abemaciclib due to its longer and more mature follow-up data and shorter treatment duration; however both agents are reasonable to consider with shared decision-making with the patient [42]. Indeed, carefully weighing patient-specific factors, such as AEs, monitoring needs, financial considerations, and preferences, ensuring the patient voice is considered through shared decision-making is recommended.

### 3.2. Practical Recommendations for Shared Decision-Making When Selecting a CDK4/6 Inhibitor

Trial eligibility should guide CDK4/6 inhibitor selection. If a patient qualifies for both abemaciclib and ribociclib, physicians should compare key differences in toxicity, dosing (daily vs. twice daily, continuous vs. intermittent), treatment duration, and impact on patients’ quality of life. Physicians should discuss efficacy, risks, and practical considerations such as AEs, monitoring, hospital visits, and financial coverage. Patients should also understand potential side effects, how to manage them, and that dose reductions, when needed, can improve treatment adherence without compromising efficacy [45,46]. We recommend a dedicated visit for discussion and questions related to CDK4/6 inhibitor selection, using easy-to-follow decision aids such as comparative tables explaining treatment outcomes, potential adverse events, and risk reduction to enhance patient understanding. Tools like simplified treatment calendars, symptom logs, and other educational materials can help patients participate in their treatment plan and feel empowered to successfully complete therapy. Early, open-ended discussions about treatment preferences (e.g., “Can you accommodate additional hospital visits?”) and concerns (e.g., “What worries you most about this treatment?”) can help personalize decisions, ensuring tailored recommendations and patient-centred care.

## 4. Practical Recommendations for the Clinical Management of CDK4/6 Inhibitors

### 4.1. Management of Common AEs Across CDK4/6 Inhibitors

To ensure consistent AE management, the most up-to-date version of the Common Terminology Criteria for Adverse Events (CTCAE) criteria should be used across all AEs, regardless of the CDK4/6 inhibitor used, enabling uniform care across the multidisciplinary team [47].

Fatigue is a common AE in patients with breast cancer, impacting their ability to return to normal activities, including work and social engagements [48]. Clear education about the multifactorial and reversible nature of fatigue, alongside practical strategies for its management, can empower patients to continue with therapy [49]. It is important to explain fatigue can be caused by the cancer itself, treatment-related side effects, and psychological factors [50]. Leveraging tools like the Canadian Association of Psychosocial Oncology (CAPO) algorithm [51] for cancer-related fatigue can help with screening and assessment to rule out reversible issues that may cause fatigue, such as anemia, hypothyroidism, and iron deficiency [50]. In the absence of an identified reversible cause, key strategies to manage fatigue include regular exercise, cognitive behavioural therapy, and mindfulness-based programs [50]. There is insufficient evidence to recommend pharmacological treatment, herbal medicines, or acupuncture to manage fatigue [50].

### 4.2. Management of Most Frequent AEs for Abemaciclib

**Figure 2:**
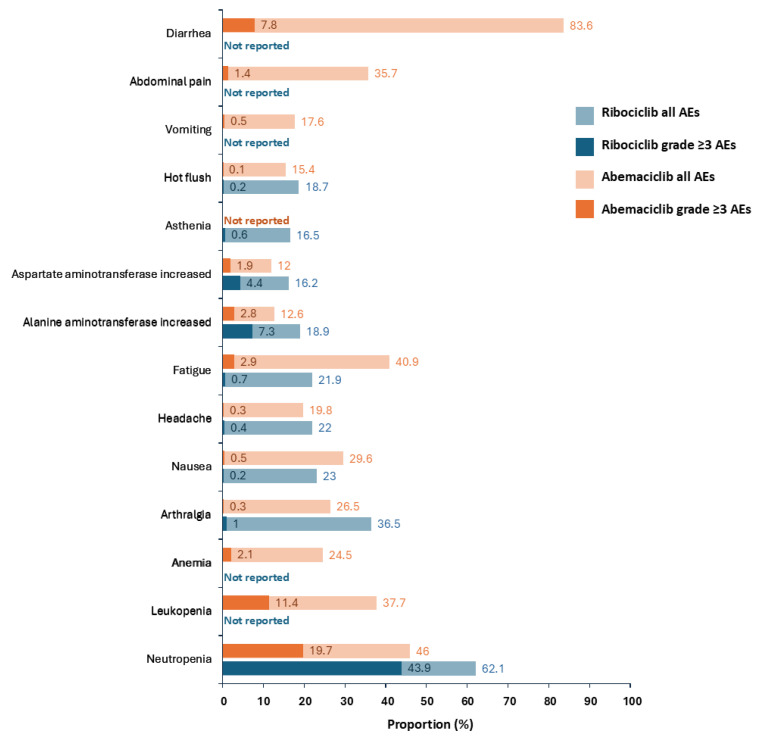
Common Adverse Events (≥15%) from the monarchE and NATALEE Trials [19,20]. AE: adverse event.

The most frequent AEs in the abemaciclib and ET arm (any grade and grade ≥ 3) in monarchE include diarrhea (83% and 8%), fatigue (41% and 3%), neutropenia (46% and 20%), leukopenia (38% and 11%), and abdominal pain (36% and 1%; Figure 2) [19]. In monarchE, 18.5% of patients discontinued treatment due to AEs; however, 68% of these patients continued ET either during treatment or in the follow-up period after stopping abemaciclib [36]. The most common AEs leading to discontinuation were diarrhea (5.3%), fatigue (2.0%) and neutropenia (0.9%); 66.8% of abemaciclib discontinuations were due to grade 1/2 AEs and not protocol mandated, highlighting the low use of dose modifications by trial investigators to manage tolerability. Further, most discontinuations occurred during the first few months, stabilizing beyond 6 months [36].

Diarrhea typically occurred early (median onset: 8 days), was short-lived (median duration for grades 2–3: 5–6 days), and was effectively managed with antidiarrheal medication and dose adjustments [19]. Given the limited evidence, we advise against prophylactic use of loperamide or probiotics to prevent abemaciclib-induced diarrhea [52,53]. Instead, we recommend informing patients that diarrhea is likely to occur when they start abemaciclib. This allows for timely interventions, such as dietary adjustments and antidiarrheal medications (with a prescription provided when the CDK4/6 inhibitor is prescribed, along with clear instructions for use), to help reduce its frequency and severity. Clinicians should grade both diarrhea and abdominal discomfort using CTCAE criteria to make necessary dose adjustments and implement appropriate symptom management strategies to ensure patient comfort and treatment adherence [47].

### 4.3. Management of Most Frequent AEs for Ribociclib

The most frequent AEs in the ribociclib and non-steroidal aromatase inhibitor (NSAI) arm (any grade and grade ≥ 3) in NATALEE include neutropenia (62% and 44%), arthralgia (37% and 1%), liver-related AEs (25% and 8%), nausea (23% and 0.2%), headache (22% and 0.4%), and fatigue (22% and 1%; Figure 2) [20]. Most ribociclib-related AEs, such as neutropenia and transaminitis, were predominantly asymptomatic laboratory findings requiring additional monitoring [20]. There have also been reports of ribociclib-induced cutaneous AEs, most commonly eczematous dermatitis and maculopapular reaction [54]. Upon CTCAE grading, applying an interdisciplinary approach involving dose modulation and dermatological interventions (e.g., topical emollients or topical/systemic steroids) can help prevent treatment discontinuation, potentially improving long-term outcomes [54].

A low incidence of drug-induced liver injury (DILI) was reported in NATALEE, (i.e., 0.3%) [20], and real-world evidence/long-term data are required to better understand these risks. A retrospective observational study of the FDA Adverse Event Reporting System (FAERS) database showed that ribociclib has a higher risk of causing liver injury compared to abemaciclib [55]. This is aligned with the pivotal trials where there were no reported cases of DILI and only 0.9% of patients discontinued abemaciclib due to elevated transaminases [36]. In NATALEE, liver-related events were the most common AE leading to discontinuation, as seen in 9% of patients in the ribociclib-NSAI arm [20]. While direct/predictable liver toxicity is dose-related and arises shortly after drug exposure, idiosyncratic or unpredictable DILI may occur with variable latency, even when taken at the recommended dose [56]. Idiosyncratic DILI can be immune-mediated or non-immune-mediated, and the immune-mediated subtype is particularly challenging to treat [57]. Dose modification and management recommendations in the Product Monograph should be followed for any hepatobiliary toxicity [33]. Furthermore, liver function tests (LFTs) should be conducted before starting ribociclib, then monitored every 2 weeks for the first 2 cycles, at the start of the next 4 cycles, and as needed. This monitoring frequency should be increased (e.g., twice weekly) if grade ≥ 2 abnormalities occur [33].

### 4.4. Management of AEs Related to the ET Backbone

It is crucial to differentiate AEs related to CDK4/6 inhibitors from those caused by concomitant ET (e.g., AIs), such as myalgia, arthralgia, fatigue, and vasomotor symptoms, to prevent unnecessary treatment interruptions [58]. In most cases, ET is started in advance of the CDK4/6 inhibitor to ensure tolerability and compliance of that agent. We recommend reviewing these symptoms with patients ahead of treatment initiation and encouraging them to keep a diary of symptoms once a CDK4/6 inhibitor is started to help differentiate any new symptoms from those caused by the ET backbone.

For patients who are intolerant to an AI, switching to a different AI or using tamoxifen is a viable option, but it should be noted tamoxifen is contraindicated with ribociclib due to increased QTc risks and potential for increased levels of CYP3A4 substrates [37]. Additionally, since tamoxifen increases the risk of venous thromboembolism (VTE), if used with abemaciclib, it is essential to discuss VTE risk reduction strategies, including smoking cessation and physical activity and alert patients of the symptoms or signs of thromboembolic disease [59]. For patients considered at high risk for a VTE, we recommend an individualized approach regarding prophylaxis/prevention in collaboration with a hematologist.

### 4.5. Implementing Dose Holds and Reductions

**Figure 3:**
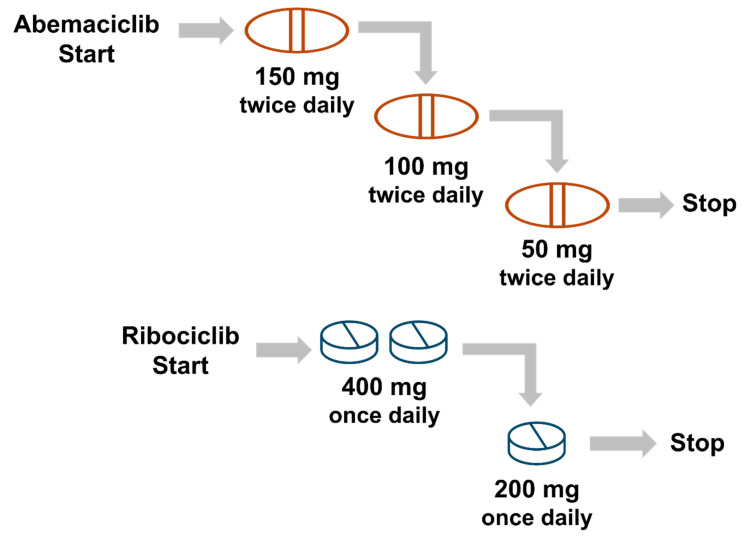
Recommended Dose Holds for Abemaciclib and Ribociclib [32,33].

Patients should be informed that dose modifications, such as holds or reductions, are commonly necessary to manage toxicities while maintaining the clinical benefits of CDK4/6 inhibitors. For abemaciclib, the recommended starting dose is 150 mg twice daily and can be reduced to 100 mg BID and/or 50 mg BID as needed for tolerability (Figure 3) [38]. For ribociclib, the recommended starting dose is 400 mg once daily with only one dose adjustment recommended, resulting in a final dose of 200 mg once daily (Figure 3) [33,39].

In monarchE, 61.7% of patients receiving abemaciclib required dose interruptions and 43.4% needed reductions due to AEs such as diarrhea, neutropenia, and fatigue [36]. Abemaciclib dose modifications occurred early during treatment and were used to effectively manage AEs, improving tolerability and minimizing treatment discontinuations [36]. Furthermore, it has been demonstrated that the efficacy of adjuvant abemaciclib is not compromised by protocol mandated dose reductions [45]. The TRADE study is currently underway to investigate a dose escalation strategy for abemaciclib based on tolerability [60]; however, until further evidence is available, we recommend following the Product Monograph by starting with 150 mg BID [32].

In NATALEE, 22.8% of patients receiving 400 mg ribociclib once daily for 3 weeks on and 1 week off required a dose reduction mostly due to liver-related AEs [46]. While full safety information, including dose interruptions or holds, is pending disclosure for NATALEE, liver function should be monitored regularly (i.e., at baseline and then every 2 weeks for the first 2 cycles, at the beginning of each of the subsequent 4 cycles, and as clinically indicated) to ensure early intervention when abnormalities are detected with ribociclib [31]. Similar to abemaciclib, a recent update from NATALEE demonstrated that the efficacy of adjuvant ribociclib is not compromised by dose reductions [46].

### 4.6. Practical Recommendations for Monitoring

**Figure 4:**
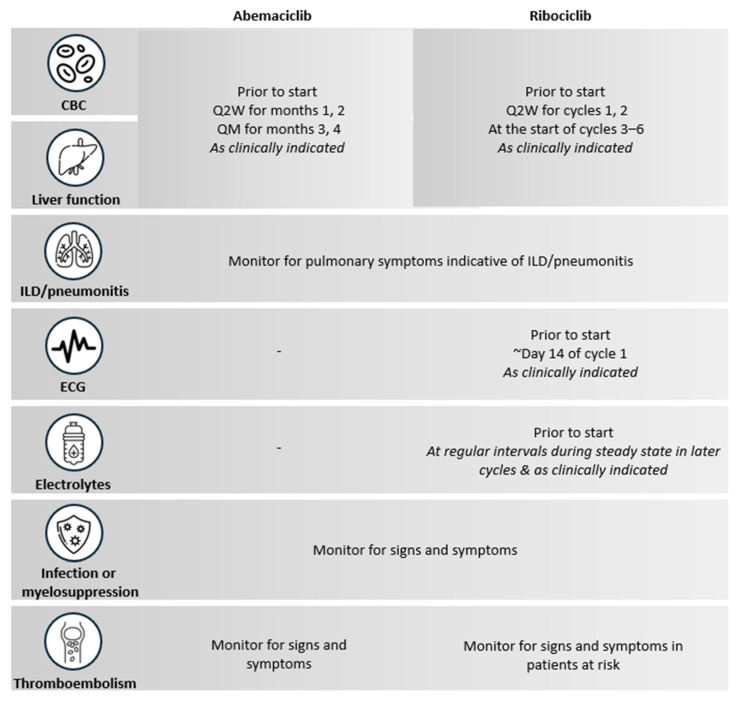
Monitoring Recommendations for Abemaciclib and Ribociclib [32,33]. CBC: complete blood count; ECG: electrocardiogram; ILD: interstitial lung disease; Q2W: every two weeks; QM: every month.

While both abemaciclib and ribociclib require monitoring, the specific parameters and schedules differ based on their distinct safety profiles and are summarized in Figure 4 [32,33].

For abemaciclib, monitoring should prioritize ongoing evaluations of complete blood count (CBC), liver enzymes, creatinine, and clinical symptoms such as diarrhea [32]. It should be noted that creatinine elevation with abemaciclib may be observed since it has a role in inhibiting renal tubular transporters, but this does not affect glomerular function, as evidenced by unchanged markers of renal function like blood urea nitrogen, cystatin C, and cystatin C-based glomerular filtration rate (GFR) [61]. Although estradiol elevation is sometimes reported, certain immunoassays may produce falsely elevated results in patients treated with abemaciclib and ovarian suppressors due to assay limitations, not the drug itself [62]. These false elevations can occur with cross-reacting substances like selective estrogen receptor degraders or AIs [63,64]. Therefore, if patients have unexpectedly high estradiol levels while on these treatments, additional measurements using highly sensitive and specific methods, such as mass spectrometry, may be conducted to ensure accuracy [65].

For ribociclib, monitoring should prioritize CBC, liver enzymes, and electrolytes, as well as electrocardiogram (ECG) assessments prior to treatment initiation, during cycle 1 at approximately day 14, at the beginning of cycle 2, and at regular intervals thereafter to evaluate QTc interval prolongation [33,37].

To manage laboratory abnormalities associated with CDK4/6 inhibitor use, clinicians should grade toxicities based on the most up-to-date version of the CTCAE criteria and implement dose adjustments per the Product Monograph [32]. For serious grade ≥ 3 toxicities, we recommend consultation with the appropriate subspecialty. As patients stabilize, follow-up intervals can be extended to every three months, incorporating streamlined approaches such as pre-visit blood work and pharmacy reviews to improve efficiency.

### 4.7. Switching Between CDK4/6 Inhibitors

Switching between CDK4/6 inhibitors may be considered under specific circumstances, such as in patients who experience intolerable toxicity with the first CDK4/6 inhibitor they are prescribed. It may also be necessary when changing the ET backbone from an AI to tamoxifen, as ribociclib is contraindicated with tamoxifen [31]. Until further publication of real-world evidence, this consensus reflects the opinion of the authors as a safe approach. In such cases, the total duration of adjuvant therapy remains unclear and may depend on the patient’s tolerability to the second agent. If the switch occurs relatively early into adjuvant treatment, the duration of therapy should mirror that of the reference publication for the second agent. Clinicians should also anticipate practical challenges, such as the financial implications of switching therapies and involve Drug Access Navigators early to expedite applications and documentation if a switch is anticipated.

In light of potential challenges of switching CDK4/6 inhibitors, patients should be reassured that dose reductions are a viable option to improve tolerability without compromising efficacy, to stay on initial treatment. Switching therapy should be reserved for serious AEs that persist despite dose reductions [45,46]. It should be noted that for patients to switch between CDK4/6 inhibitors, they must meet the eligibility criteria for both agents based on their respective Phase III clinical trials [19,20].

### 4.8. Optimizing Multidisciplinary Collaboration During Monitoring and Follow-Up

NPs and pharmacists can oversee routine follow-ups, identify early signs of toxicity, and provide timely interventions. Alternative models of care can also be implemented to help alleviate resource and time burden faced by many clinics. A nurse-led supportive care program for women with newly diagnosed breast cancer demonstrated significant improvement of quality of life and symptom management during adjuvant chemotherapy [23]. In this randomized controlled pilot study, patients in the intervention group showed significantly higher global health and functional status scores, and lower symptom burden compared to controls [23]. Furthermore, a pharmacist-led virtual clinic for patients with breast cancer on CDK4/6 inhibitors improved adherence to laboratory testing and the patient-reported adherence to the prescribed medication was more than 99% [24]. In this model, patients received structured follow-ups every 2 weeks initially, then monthly, focusing on lab review, adherence, AE monitoring, and drug interactions [24]. The clinic identified 38 potential drug therapy issues, leading to targeted interventions such as patient education, symptom management, and QT monitoring [24].

Emerging technologies can also be leveraged to address logistical challenges associated with monitoring requirements, such as the FDA-approved KardiaMobile^®^ app to simplify QTc monitoring and reduce the burden of frequent ECG assessments [66]. Other useful tools that can be leveraged include validated QTcF calculators or pill reminder applications.

### 4.9. Tools and Tips for Patient Discussions

A summary checklist of strategies to optimize shared decision-making between physicians and patients is presented in Table 2. Clinicians and patients may choose to supplement care with digital patient platforms, which can provide additional support and resources. Given the variability in available tools, clinician preferences, and patient access, the use of these platforms should be individualized. While not essential for all, they can enhance patient engagement, streamline communication, and support ongoing monitoring where appropriate.

## Figures and Tables

**Table 1:** Summary of Key Differences Between Abemaciclib and Ribociclib *.

Parameter	Abemaciclib [19,32]	Ribociclib [20,33]	Key Takeaway
Phase III Trial Eligibility Criteria	**monarchE** Cohort 1: • ≥4 positive ALN **OR** • 1–3 positive ALN and at least 1 of: ○ Grade 3 disease ○ Tumour size ≥ 5 cm Cohort 2: • 1–3 positive ALN and Ki-67 ≥ 20% • Tumour grade below 3 • Tumour size < 5 cm *In monarchE, microscopic and macroscopic nodal involvement were allowed*	**NATALEE** • Patients with stage IIB or III disease were eligible irrespective of nodal status. • Patients with stage IIA disease were eligible if: ○ ≥1 lymph node involved ○ No nodal involvement and grade 3 tumours. ○ No nodal involvement and a grade 2 tumour with: ▪ Ki-67 ≥ 20% ▪ High genomic risk score (Oncotype DX Breast RS, MammaPrint^®^, EndoPredict^®^)	Abemaciclib may be considered for “higher-risk patients” given the requirement for node-positive disease in monarchE.
ET Partner	AI or tamoxifen †	NSAI	Abemaciclib may be considered with tamoxifen for patients who cannot tolerate AIs. †
iDFS in Clinical Trial Overall Population	5-year iDFS [34] Abemaciclib + ET: 83.6% ET alone: 76.0% HR: 0.68 (95% CI: 0.60, 0.77), Nominal *p* < 0.001 **7.6%** absolute increase in iDFS with **addition of abemaciclib** to ET.	4-year iDFS Ribociclib + NSAI: 88.5% NSAI alone: 83.6% HR: 0.72 (95% CI: 0.61, 0.84), *p* < 0.0001 [21] **4.9%** absolute increase in iDFS with **addition of ribociclib** to NSAI.	Both abemaciclib and ribociclib demonstrate iDFS benefits in the overall population and across key subgroups [35].
Overall Survival	Not reported	Not reported	Overall survival data remain immature for both abemaciclib and ribociclib.
Data Maturity ^‡^	5 years	4 years	Abemaciclib has greater data maturity/longer-term follow-up.
Treatment Length	2 years	3 years	A longer treatment duration may extend the burden of toxicity and impact quality of life and/or adherence.
Dosing Regimen	150 mg PO BID	400 mg PO once daily for 21 days, then 7 days off treatment	Scheduling and duration may impact patient preference and adherence.
Recommended Dose Reductions	Reduction 1 to 100 mg PO BID Reduction 2 to 50 mg PO BID	Reduction to 200 mg PO once daily	Both agents can be dose reduced for toxicity.
Most Frequent AEs (All Grades; **Grade ≥ 3**)	• Diarrhea (83.6%; **7.8%)** • Neutropenia (46%; **19.7%**) • Fatigue (40.9%; **2.9%**) • Leukopenia (37.7%; **11.4%**) • Abdominal pain (35.7%; **1.4%**)	• Neutropenia (62.1%; **43.9%**) • Arthralgia (36.5%; **1.0%**) • Liver-related events (25.4%; **8.3%**) • Nausea (23.0%; **0.2%**) • Headache (22.0%; **0.4%**) • Fatigue (21.9%; **0.7%**)	Diarrhea with abemaciclib + ET typically occurred early, was short-lived, and was effectively managed with antidiarrheal medication and dose adjustments [19]. Most ribociclib-related AEs, such as neutropenia and liver-related events, were predominantly asymptomatic laboratory findings requiring additional monitoring [20].
AEs Leading to Treatment Discontinuation (%) [36]	• Diarrhea (5.3%) • Fatigue (**2.0%**) • Neutropenia (**0.9%**)	• Liver-related events (**8.9%**) • Arthralgia (**1.3%**)	
Product Monograph Recommended Monitoring	• Prior to initiation, Q2W for first 2 months, QM for next 2 months, as clinically indicated: ○ CBC ○ Liver function • ILD/pneumonitis • Infection/myelosuppression • Thromboembolism (especially in patients receiving concomitant tamoxifen)	• Prior to initiation, Q2W for first 2 cycles, at the beginning of each of the subsequent 4 cycles, as clinically indicated: ○ CBC ○ Liver function • Prior to initiation, during cycle 1 ~D14, beginning of cycle 2, regular intervals thereafter and whenever clinically indicated: ○ ECG • Prior to initiation, then at regular intervals and whenever clinically indicated: ○ Electrolytes • ILD/pneumonitis • Thromboembolism (in those at risk)	Treatment monitoring should follow the Product Monograph for both agents.
DDI Considerations	Metabolic/Transport Effects • CYP3A4 (major), BCRP/ABCG2 (minor), P-glycoprotein/ABCB1 (minor) [37] Avoid Using With • CYP3A inhibitors/inducers	Metabolic/Transport Effects • Substrate of CYP3A4 (major); inhibits CYP3A4 (moderate) [38] • Possible risk of TdP [39] Avoid Using With • CYP3A inhibitors/inducers • Anti-arrhythmic medicines/drugs that prolong the QT interval (e.g., tamoxifen) • Drugs that decrease electrolyte levels • Tamoxifen	Ribociclib requires monitoring for QTc interactions (e.g., with antidepressants, antibiotics), or with drugs affecting electrolyte levels [33].

* Note: Data presented in the table are from the monarchE (abemaciclib) and NATALEE (ribociclib) trials; without the availability of a head-to-head trial, cross-trial comparisons should be avoided; † Experts recommend abemaciclib be administered with an AI (steroid or non-steroidal) given the higher VTE risk associated with tamoxifen [40]; ^‡^ Longer-term data follow-up expected to be reported in May 2025.ABCB1: ATP-binding cassette subfamily B member 1; ABCG2: ATP-binding cassette super-family G 2; AE: adverse event; AI: aromatase inhibitor; ALN: axillary lymph node; BCRP: breast cancer resistance protein; BID: twice daily; CBC: complete blood count; CI: confidence interval; CYP3A: cytochrome P450 family 3 subfamily A; DDI: drug–drug interaction; DILI: drug-induced liver injury; ECG: electrocardiogram; ET: endocrine therapy; HR: hazard ratio; iDFS: invasive disease-free survival; ILD: interstitial lung disease; NSAI: non-steroidal aromatase inhibitor; Q2W: every 2 weeks; QM: every month; QTc: corrected QT interval; RS: recurrence score; TdP: Torsades de Pointes; VTE: venous thromboembolism.

**Table 2:** Checklist to Optimize Shared Decision-making with Patients Across the Early Breast Cancer Patient Journey.

Diagnosis and Risk Assessment	
Once patient is identified as high risk, explain: ▯ Their elevated recurrence risk ▯ Why adding a CDK4/6 inhibitor to ET may benefit them	*Expert tip:* ✓ Use plain language and tailored messages ✓ Leverage visual aids, videos, and summaries
**Treatment decision-making**	
Adopt a collaborative and individualized approach: ▯ Explain breast cancer treatment involves multiple steps (neoadjuvant, adjuvant) ▯ Briefly introduce all possible agents upfront (ET, CDK4/6 inhibitors, chemotherapy, radiation therapy, bisphosphonates, PARP inhibitors) ▯ Outline benefits, risks, and side effects of each treatment ▯ Set expectations for monitoring and long-term management (e.g., frequency of visits and geographic proximity to centre)	*Expert tip:* ✓ Prepare patients early for additional treatments (e.g., bisphosphonates, adjuvant CDK4/6 inhibitors) ✓ Use open-ended questions to assess willingness to manage side effects and adhere to treatment ✓ Reassure patients that side effects can be managed, dose reductions are possible, and stopping treatment isn’t the only option
**Treatment initiation and follow-up**	
Ensure patients feel informed and supported: ▯ Provide a simple, high-level overview of treatment and side effect management (beyond handouts) ▯ Clarify why each drug is recommended, including side effects, monitoring, and financial considerations	*Expert tip:* ✓ Ask patients at each visit if they feel ready to continue—encourage honest feedback and shared decision-making ✓ When possible, involve pharmacists with expertise in oral oncology drugs

CDK4/6: cyclin-dependent kinase 4/6; ET: endocrine therapy; PARP: poly (ADP-ribose) polymerase.

## References

[1] Salvo E.M., Ramirez A.O., Cueto J., Law E.H., Situ A., Cameron C., Samjoo I.A. (2021). Risk of recurrence among patients with HR-positive, HER2-negative, early breast cancer receiving adjuvant endocrine therapy: A systematic review and meta-analysis. Breast.

[2] Pan H., Gray R., Braybrooke J., Davies C., Taylor C., McGale P., Peto R., Pritchard K.I., Bergh J., Dowsett M. (2017). 20-Year Risks of Breast-Cancer Recurrence after Stopping Endocrine Therapy at 5 Years. N. Engl. J. Med..

[3] Colleoni M., Sun Z., Price K.N., Karlsson P., Forbes J.F., Thurlimann B., Gianni L., Castiglione M., Gelber R.D., Coates A.S. (2016). Annual Hazard Rates of Recurrence for Breast Cancer During 24 Years of Follow-Up: Results From the International Breast Cancer Study Group Trials I to V. J. Clin. Oncol..

[4] Early Breast Cancer Trialists’ Collaborative Group (2024). Reductions in recurrence in women with early breast cancer entering clinical trials between 1990 and 2009: A pooled analysis of 155 746 women in 151 trials. Lancet..

[5] The ASCO Post. Impact of Menopausal Status on Long-TermBenefit From Antihormonal Treatment inWomen With Breast Cancer. https://ascopost.com/news/december-2024/impact-of-menopausal-status-on-long-term-benefit-from-antihormonal-treatment-in-women-with-breast-cancer/#:~:text=New%20research%20has%20shown%20that,not%20yet%20gone%20through%20menopause.

[6] Beadle B.M., Woodward W.A., Buchholz T.A. (2011). The impact of age on outcome in early-stage breast cancer. Semin. Radiat. Oncol..

[7] Copson E.R., Abraham J.E., Braybrooke J.P., Cameron D., McIntosh S.A., Michie C.O., Okines A.F.C., Palmieri C., Raja F., Roylance R. (2023). Expert UK consensus on the definition of high risk of recurrence in HER2-negative early breast cancer: A modified Delphi panel. Breast.

[8] Martei Y.M., Matro J.M. (2015). Identifying patients at high risk of breast cancer recurrence: Strategies to improve patient outcomes. Breast Cancer.

[9] Sheffield K.M., Peachey J.R., Method M., Grimes B.R., Brown J., Saverno K., Sugihara T., Cui Z.L., Lee K.T. (2022). A real-world US study of recurrence risks using combined clinicopathological features in HR-positive, HER2-negative early breast cancer. Future Oncol..

[10] Tolaney S.M., Sammons S., Cortes J., Liepa A., Sugihara T., Cui Z., Gathirua-Mwangi W., Grimes B., Shahir A., Monaco M. Real-world Risk of Recurrence by Nodal Status in Patients with HR+, HER2-, Node-positive, High-risk Early Breast Cancer. Presented at San Antonio Breast Cancer Symposium (SABCS) 47th Annual Meeting.

[11] Gonzalez-Hurtado D., Rivero S., Samame Perez-Vargas J.C., Petracci F.E. (2023). Hormone Receptor-Positive/HER2-Negative Early Breast Cancer High-Risk Population: An Algorithm for Optimization Systemic Adjuvant Treatment Based on 2022 Updates. Breast Cancer.

[12] Kalinsky K., Barlow W.E., Gralow J.R., Meric-Bernstam F., Albain K.S., Hayes D.F., Lin N.U., Perez E.A., Goldstein L.J., Chia S.K.L. (2021). 21-Gene Assay to Inform Chemotherapy Benefit in Node-Positive Breast Cancer. N. Engl. J. Med..

[13] Sparano J.A., Gray R.J., Makower D.F., Pritchard K.I., Albain K.S., Hayes D.F., Geyer C.E., Dees E.C., Goetz M.P., Olson J.A. (2018). Adjuvant Chemotherapy Guided by a 21-Gene Expression Assay in Breast Cancer. N. Engl. J. Med..

[14] Cardoso F., van’t Veer L.J., Bogaerts J., Slaets L., Viale G., Delaloge S., Pierga J.Y., Brain E., Causeret S., DeLorenzi M. (2016). 70-Gene Signature as an Aid to Treatment Decisions in Early-Stage Breast Cancer. N. Engl. J. Med..

[15] Finn R.S., Martin M., Rugo H.S., Jones S., Im S.A., Gelmon K., Harbeck N., Lipatov O.N., Walshe J.M., Moulder S. (2016). Palbociclib and Letrozole in Advanced Breast Cancer. N. Engl. J. Med..

[16] Turner N.C., Slamon D.J., Ro J., Bondarenko I., Im S.A., Masuda N., Colleoni M., DeMichele A., Loi S., Verma S. (2018). Overall Survival with Palbociclib and Fulvestrant in Advanced Breast Cancer. N. Engl. J. Med..

[17] Hortobagyi G.N., Stemmer S.M., Burris H.A., Yap Y.S., Sonke G.S., Hart L., Campone M., Petrakova K., Winer E.P., Janni W. (2022). Overall Survival with Ribociclib plus Letrozole in Advanced Breast Cancer. N. Engl. J. Med..

[18] Goetz M.P., Toi M., Campone M., Sohn J., Paluch-Shimon S., Huober J., Park I.H., Trédan O., Chen S.C., Manso L. (2017). MONARCH 3: Abemaciclib As Initial Therapy for Advanced Breast Cancer. J. Clin. Oncol..

[19] Johnston S.R.D., Toi M., O’Shaughnessy J., Rastogi P., Campone M., Neven P., Huang C.S., Huober J., Jaliffe G.G., Cicin I. (2023). Abemaciclib plus endocrine therapy for hormone receptor-positive, HER2-negative, node-positive, high-risk early breast cancer (monarchE): Results from a preplanned interim analysis of a randomised, open-label, phase 3 trial. Lancet Oncol..

[20] Slamon D., Lipatov O., Nowecki Z., McAndrew N., Kukielka-Budny B., Stroyakovskiy D., Yardley D.A., Huang C.S., Fasching P.A., Crown J. (2024). Ribociclib plus Endocrine Therapy in Early Breast Cancer. N. Engl. J. Med..

[21] Fasching P.A., Stroyakovskiy D., Yardley D., Huang C.S., Crown J.P., Bardia A., Chia S., Im S.A., Martin Jimenez M., Xu B. (2024). LBA13 Adjuvant ribociclib (RIB) plus nonsteroidal aromatase inhibitor (NSAI) in patients (Pts) with HR+/HER2− early breast cancer (EBC): 4-year outcomes from the NATALEE trial. Ann. Oncol..

[22] Kanjanapan Y., Anderson W., Smith M., Green J., Chalker E., Craft P. (2024). Real-World Analysis of Breast Cancer Patients Qualifying for Adjuvant CDK4/6 Inhibitors. Clin. Breast Cancer.

[23] Kucuk B.Y., Bahceli P.Z. (2024). The Effects of Nurse-Led Supportive CareProgram on Quality of Life in Women withBreast Cancer Receiving AdjuvantChemotherapy: A Randomized ControlledPilot Study. Semin. Oncol. Nurs..

[24] Taraba J., Golbach A., Smith M., Mara K., Giridhar K. (2022). Development and Implementation of a Pharmacist-Led Virtual Clinic Improve the Management of Patients with Metastatic Breast Cancer Receiving CDK4/6 Inhibitors. JHOP.

[25] Beck A.C., Morrow M. (2023). Axillary lymph node dissection: Dead or still alive?. Breast.

[26] Rocco N., Ghilli M., Curcio A., Bortul M., Burlizzi S., Cabula C., Cabula R., Ferrari A., Folli S., Fortunato L. (2024). Is routine axillary lymph node dissection needed to tailor systemic treatments for breast cancer patients in the era of molecular oncology? A position paper of the Italian National Association of Breast Surgeons (ANISC). Eur. J. Surg. Oncol..

[27] Jankowski C., Houvenaeghel G., Renaudeau C., Leveque J., Marchal F., Benbara A., Barranger E., Rouzier R., Cohen M., Classe J.-M. (2024). How can we optimize the surgical management ofthe axilla in breast cancer since the MonarchE trial?. Breast Cancer Res. Treat..

[28] de Boniface J., Appelgren M., Szulkin R., Alkner S., Andersson Y., Bergkvist L., Frisell J., Gentilini O.D., Kontos M., Kuhn T. (2024). Completion axillary lymph node dissection for the identification of pN2-3 status as an indication for adjuvant CDK4/6 inhibitor treatment: A post-hoc analysis of the randomised, phase 3 SENOMAC trial. Lancet Oncol..

[29] Gentilini O.D., Botteri E., Sangalli C., Galimberti V., Porpiglia M., Agresti R., Luini A., Viale G., Cassano E., Peradze N. (2023). Sentinel Lymph Node Biopsy vs No Axillary Surgery in Patients With Small Breast Cancer and Negative Results on Ultrasonography of Axillary Lymph Nodes: The SOUND Randomized Clinical Trial. JAMA Oncol..

[30] Reimer T., Stachs A., Veselinovic K., Kuhn T., Heil J., Polata S., Marme F., Muller T., Hildebrandt G., Krug D. (2025). Axillary Surgery in Breast Cancer—Primary Results of the INSEMA Trial. N. Engl. J. Med..

[31] Slamon D.J., Fasching P.A., Hurvitz S., Chia S., Crown J., Martin M., Barrios C.H., Bardia A., Im S.A., Yardley D.A. (2023). Rationale and trial design of NATALEE: A Phase III trial of adjuvant ribociclib + endocrine therapy versus endocrine therapy alone in patients with HR+/HER2- early breast cancer. Ther. Adv. Med. Oncol..

[32] Eli Lilly Canada Inc (2023). VERZENIO (abemaciclib) Health Canada Product Monograph.

[33] Pharmaceuticals Canada Inc (2025). KISQALI (ribociclib) Health Canada Product Monograph.

[34] Rastogi P., O’Shaughnessy J., Martin M., Boyle F., Cortes J., Rugo H.S., Goetz M.P., Hamilton E.P., Huang C.S., Senkus E. (2024). Adjuvant Abemaciclib Plus Endocrine Therapy for Hormone Receptor-Positive, Human Epidermal Growth Factor Receptor 2-Negative, High-Risk Early Breast Cancer: Results From a Preplanned monarchE Overall Survival Interim Analysis, Including 5-Year Efficacy Outcomes. J. Clin. Oncol..

[35] Keskinkilic M., Arayici M.E., Basbinar Y., Ellidokuz H., Yavuzsen T., Oztop I. (2024). The efficacy and safety of CDK4/6 inhibitors combined with endocrine therapy versus endocrine therapy alone in the adjuvant treatment of patients with high-risk invasive HR+/HER2-early breast cancer: A comprehensive updated meta-analysis of randomized clinical trials. Breast.

[36] Rugo H.S., O’Shaughnessy J., Boyle F., Toi M., Broom R., Blancas I., Gumus M., Yamashita T., Im Y.H., Rastogi P. (2022). Adjuvant abemaciclib combined with endocrine therapy for high-risk early breast cancer: Safety and patient-reported outcomes from the monarchE study. Ann. Oncol..

[37] UpToDate Lexidrug/Abemaciclib (2025). UpToDate Lexidrug App, version 8.2.0.

[38] UpToDate Lexidrug/Ribociclib (2025). UpToDate Lexidrug App, version 8.2.0.

[39] Woosley, R.L.; HC; Gallo, T.; Woosley, R.D.; Lambson, J.; Romero, K.A. QTdrugs List. www.CredibleMeds.org.

[40] Matthews A., Stanway S., Farmer R.E., Strongman H., Thomas S., Lyon A.R., Smeeth L., Bhaskaran K. (2018). Long term adjuvant endocrine therapy and risk of cardiovascular disease in female breast cancer survivors: Systematic review. BMJ.

[41] Chlebowski R.T., Kim J., Haque R. (2014). Adherence to endocrine therapy in breast cancer adjuvant and prevention settings. Cancer Prev. Res..

[42] Freedman R.A., Caswell-Jin J.L., Hassett M., Somerfield M.R., Giordano S.H., Optimal Adjuvant C. (2024). Targeted Therapy for Early Breast Cancer Guideline Expert, P. Optimal Adjuvant Chemotherapy and Targeted Therapy for Early Breast Cancer-Cyclin-Dependent Kinase 4 and 6 Inhibitors: ASCO Guideline Rapid Recommendation Update. J. Clin. Oncol..

[43] monarchE: Protocol I3YMCJPCF(e) A Randomized, Open Label, Phase 3 Study of Abemaciclib Combined with Standard Adjuvant Endocrine Therapy versus Standard Adjuvant Endocrine Therapy Alone in Patients with High Risk, Node Positive, Early Stage, Hormone Receptor Positive, Human Epidermal Receptor 2 Negative, Breast Cance. https://cdn.clinicaltrials.gov/large-docs/97/NCT03155997/Prot_000.pdf.

[44] Agostinetto E., Arecco L., de Azambuja E. (2024). Adjuvant CDK4/6 Inhibitors for Early Breast Cancer: How to Choose Wisely?. Oncol. Ther..

[45] Goetz M.P., Cicin I., Testa L., Tolaney S.M., Huober J., Guarneri V., Johnston S.R.D., Martin M., Rastogi P., Harbeck N. (2024). Impact of dose reductions on adjuvant abemaciclib efficacy for patients with high-risk early breast cancer: Analyses from the monarchE study. NPJ Breast Cancer.

[46] Barrios C.H., Harbeck N., Hortobagyi G., O’Shaughnessy J., Huang C.S., Jimenez M.M., Juric D., Pistilli B., Xu B., De Laurentiis M. NATALEE update: Safety and treatment duration of ribociclib + nonsteroidal aromatase inhibitor in patients with HR+/HER2− early breast cancer. Presented at ESMO Breast Cancer Annual Congress.

[47] (2017). Common Terminology Criteria for Adverse Events (CTCAE).

[48] National Cancer Institute. Cancer Fatigue. https://www.cancer.gov/about-cancer/treatment/side-effects/fatigue.

[49] National Cancer Institute. Fatigue (PDQ®)–Health Professional Version. https://www.cancer.gov/about-cancer/treatment/side-effects/fatigue/fatigue-hp-pdq.

[50] Bower J.E., Lacchetti C., Alici Y., Barton D.L., Bruner D., Canin B.E., Escalante C.P., Ganz P.A., Garland S.N., Gupta S. (2024). Management of Fatigue in Adult Survivors of Cancer: ASCO-Society for Integrative Oncology Guideline Update. J. Clin. Oncol..

[51] Canadian Association of Psychosocial Oncology (CAPO). Screening and Assessment—Cancer-Related Fatigue in Adults with Cancer. https://www.capo.ca/resources/Documents/Guidelines/5.%20A%20Pan%20Canadian%20Practice%20Guideline%20for%20Screening,%20Assessment,%20and%20Management%20of%20Cancer-Related%20Fatigue%20in%20Adults.pdf.

[52] Hamilton E., Cortes J., Ozyilkan O., Chen S.C., Petrakova K., Manikhas A., Jerusalem G., Hegg R., Huober J., Chapman S.C. (2021). nextMONARCH: Abemaciclib Monotherapy or Combined With Tamoxifen for Metastatic Breast Cancer. Clin. Breast Cancer.

[53] Masuda H., Tanabe Y., Sakai H., Matsumoto K., Shimomura A., Doi M., Miyoshi Y., Takahashi M., Sagara Y., Tokunaga S. (2023). Efficacy of probiotics and trimebutine maleate for abemaciclib-induced diarrhea: A randomized, open-label phase II trial (MERMAID, WJOG11318B). Breast.

[54] Borroni R.G., Bartolini M., Gaudio M., Jacobs F., Benvenuti C., Gerosa R., Tiberio P., Manara S., Solferino A., Santoro A. (2024). Ribociclib-Induced Cutaneous Adverse Events in Metastatic HR+/HER2- Breast Cancer: Incidence, Multidisciplinary Management, and Prognostic Implication. Oncologist.

[55] She Y., Guo Z., Zhai Q., Liu J., Du Q., Zhang Z. (2024). CDK4/6 inhibitors in drug-induced liver injury: A pharmacovigilance study of the FAERS database and analysis of the drug-gene interaction network. Front. Pharmacol..

[56] Katarey D., Verma S. (2016). Drug-induced liver injury. Clin. Med..

[57] Verma S., Kaplowitz N. (2009). Diagnosis, management and prevention of drug-induced liver injury. Gut.

[58] Cella D., Fallowfield L.J. (2008). Recognition and management of treatment-related side effects for breast cancer patients receiving adjuvant endocrine therapy. Breast Cancer Res. Treat..

[59] Lutsey P.L., Zakai N.A. (2023). Epidemiology and prevention of venous thromboembolism. Nat. Rev. Cardiol..

[60] NCT06001762. NCT06001762.

[61] Chappell J.C., Turner P.K., Pak Y.A., Bacon J., Chiang A.Y., Royalty J., Hall S.D., Kulanthaivel P., Bonventre J.V. (2019). Abemaciclib Inhibits Renal Tubular Secretion Without Changing Glomerular Filtration Rate. Clin. Pharmacol. Ther..

[62] Kessler A.J., Patel R., Gallagher E.J., Shao T., Fasano J. (2023). Discrepancies in estradiol levels in a premenopausal woman receiving abemaciclib despite ovarian function suppression and bilateral salpingo-oophorectomy. Curr. Probl. Cancer Case Rep..

[63] Owen L.J., Monaghan P.J., Armstrong A., Keevil B.G., Higham C., Salih Z., Howell S. (2019). Oestradiol measurement during fulvestrant treatment for breast cancer. Br. J. Cancer.

[64] Mandic S., Kratzsch J., Mandic D., Debeljak Z., Lukic I., Horvat V., Gaudl A., Seric V. (2017). Falsely elevated serum oestradiol due to exemestane therapy. Ann. Clin. Biochem..

[65] Rosner W., Hankinson S.E., Sluss P.M., Vesper H.W., Wierman M.E. (2013). Challenges to the measurement of estradiol: An endocrine society position statement. J. Clin. Endocrinol. Metab..

[66] Emmett A., Kent B., James A., March-McDonald J. (2024). Evaluating the use of the mobile electrocardiogram technology KardiaMobile™ in community settings: An online survey. Nurs. Open.

